# Drying-Mediated Self-Assembly of Graphene for Inkjet Printing of High-Rate Micro-supercapacitors

**DOI:** 10.1007/s40820-020-0368-8

**Published:** 2020-01-27

**Authors:** Szymon Sollami Delekta, Mika-Matti Laurila, Matti Mäntysalo, Jiantong Li

**Affiliations:** 1 School of Electrical Engineering and Computer Science, KTH Royal Institute of Technology, Electrum 229, 16440 Kista, Sweden; 2 Laboratory for Future Electronics, Faculty of Information Technology and Communication Sciences, Tampere University, 33720 Tampere, Finland

**Keywords:** High-rate micro-supercapacitor, 3D micro-supercapacitor, Drying-mediated self-assembly, Graphene, Inkjet printing

## Abstract

Scalable fabrication of high-rate micro-supercapacitors (MSCs) is highly desired for on-chip integration of energy storage components. By virtue of the special self-assembly behavior of 2D materials during drying thin films of their liquid dispersion, a new inkjet printing technique of passivated graphene micro-flakes is developed to directly print MSCs with 3D networked porous microstructure. The presence of macroscale through-thickness pores provides fast ion transport pathways and improves the rate capability of the devices even with solid-state electrolytes. During multiple-pass printing, the porous microstructure effectively absorbs the successively printed inks, allowing full printing of 3D structured MSCs comprising multiple vertically stacked cycles of current collectors, electrodes, and sold-state electrolytes. The all-solid-state heterogeneous 3D MSCs exhibit excellent vertical scalability and high areal energy density and power density, evidently outperforming the MSCs fabricated through general printing techniques.

**Electronic supplementary material:**

The online version of this article (10.1007/s40820-020-0368-8) contains supplementary material, which is available to authorized users.

## Highlights

- Based on the drying-mediated self-assembly behavior of passivated graphene, a new kind of 2D micro-flake inks is developed to directly print high-resolution patterns with multiscale porous microstructure.
- The new ink allows to directly print complex 3D structures comprising multiple layers of heterogeneous materials.
- High-rate all-solid-state 3D micro-supercapacitors have been fully inkjet-printed with an areal capacitance surpassing 10 mF cm^−2^ at a high scan rate of 1 V s^−1^.

**Electronic supplementary material:**

The online version of this article (10.1007/s40820-020-0368-8) contains supplementary material, which is available to authorized users.

## Graphical abstract

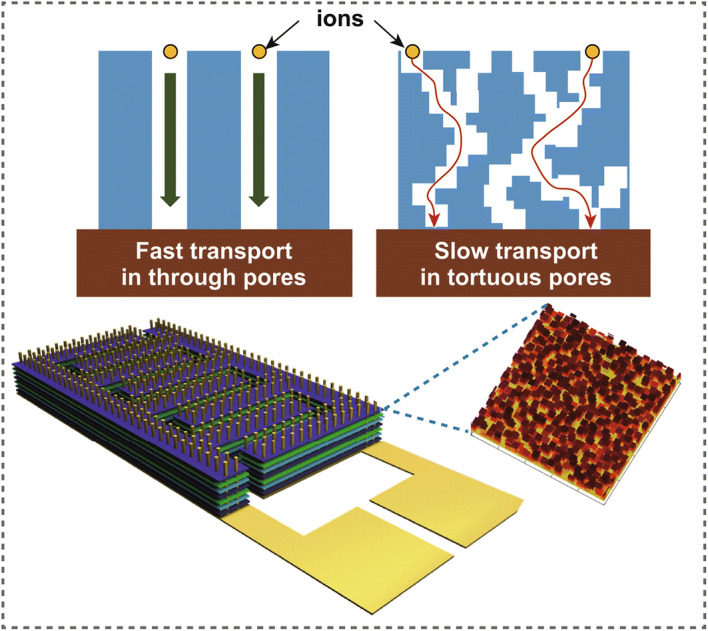

## Introduction

Over the past decade, planar micro-supercapacitors (MSCs) have attracted tremendous research interest as compact energy storage components that can be directly integrated with silicon-based electronics on chip to enable miniaturized self-powered systems [1–4]. To date, a variety of approaches have been developed to pattern versatile active materials in submillimeter feature size to fabricate MSCs of footprint area < 1 cm^2^ [1]. However, the practical on-chip integration is hindered by the lack of a comprehensive technology that simultaneously possesses simple processing, good scalability, and compatibility with micro-fabrication in silicon technology, capability of fabricating thick and multiscale porous electrodes (for sufficient energy capacity), and exemption from liquid-state electrolytes for high performance (for easy encapsulation). Among various fabrication techniques, inkjet printing is promising for on-chip integration due to its unique combination of many merits such as direct (mask-free) patterning, non-contact processing, high resolution, excellent scalability, and versatile compatibility with various substrates and active materials (especially the emerging 2D materials such as graphene [5] and MXenes [6]). As a matter of fact, it has been extensively employed to fabricate high-performance non-silicon-based electronic devices [7–10]. However, the conventional inkjet printing is only suitable for fabricating ultra-thin layers, which restricts its application in MSCs that often require “thick patterns” at sub-mm scale. In spite of a number of attempts, the printed MSCs suffer from either large footprint area [11, 12] or inferior energetic performance, e.g., low areal capacitance (< 2 mF cm^−2^) and low rate capability (limited by scan rates < 100 mV s^−1^) [13–15]. Here, by virtue of the unique self-assembly behavior of 2D materials, we develop a new kind of micro-flake inks of pseudocapacitive nanoparticle-passivated graphene to directly print sub-mm patterns with multiscale porous microstructure and large thickness of over 10 microns. In particular, the micro-flake inks enable us to fully print 3D structured MSCs which comprise multiple vertically stacked cycles of current collectors, electrodes, and gel electrolytes. The electrochemical performance of such heterogeneous 3D devices scales with the cycle number (or device thickness). Remarkably, an areal capacitance surpassing 10 mF cm^−2^ has been achieved at a high scan rate of 1 V s^−1^ for the “all-solid-state” 3D MSCs.

## Experimental Section

### 3D Modeling of Drying-Mediated Self-Assembly of 2D Materials

The 3D coarse-grained lattice gas model [16] developed by Rabani et al. is adapted to study the self-assembly behavior of graphene (or in general 2D materials) micro-flakes. In the model, the liquid thin film is represented as a 3D lattice comprising cubic cells. All the cells have size of 1 nm (comparable to the typical correlation length of a solvent) and initially are occupied exclusively by solvent, substrate (only in the lowest layer) or a micro-flake. One micro-flake is 1 nm thick and can span multiple cells depending on its lateral size *L*_*m*_. For simplicity, in this work all the micro-flakes are in square shape. Each cell *i* accompanies with three binary variables, *l*_*i*_, *n*_*i*_, and *s*_*i*_, to indicate the presence (of value 1) or absence (of value 0) of liquid, micro-flake, and substrate, respectively. During the drying process, the *solvent cell fluctuates* between liquid (*l*_*i*_ = 1) and gas (*l*_*i*_ = 0) states (to mimic the solvent evaporation or condensation), while the *micro*-*flakes move* in randomly chosen directions. A liquid cell *i* can evaporate (*l*_*i*_ changes from 1 to 0) only if at least one of its adjacent lattice cells *j* contains vapor, i.e., *l*_*j*_ + *n*_*j*_ + *s*_*j*_ = 0. A gas cell *i* can condense (*l*_*i*_ changes from 0 to 1) only if at least one but not all of its adjacent lattice cells *j* satisfy *l*_*j*_ + *n*_*j*_ + *s*_*j*_ = 0. The constraints ensure that the evaporation takes place layer by layer (though in each layer the evaporation can be inhomogeneous), and avoid the formation of bubbles (i.e., boiling) and fully vapor-surrounded liquid or micro-flakes [16]. For simplicity, only the translational motion of the micro-flakes is considered, but not their rotational motion. Therefore, during the motion, a micro-flake is displaced by a single lattice spacing in a randomly chosen direction (up, down, left, right, forward, or backward). But a micro-flake can only move into the region which is fully filled with liquid, and after the micro-flake moves, the left void cells are replenished by liquid. This constraint mimics the very low mobility of micro-flakes at a dry surface. In other words, with the liquid evaporation, the micro-flakes become frozen [17]. Therefore, the model includes explicitly the dynamics of solvent evaporation and micro-flake diffusion, and their mutual impact on each other.

**Fig. 1:**
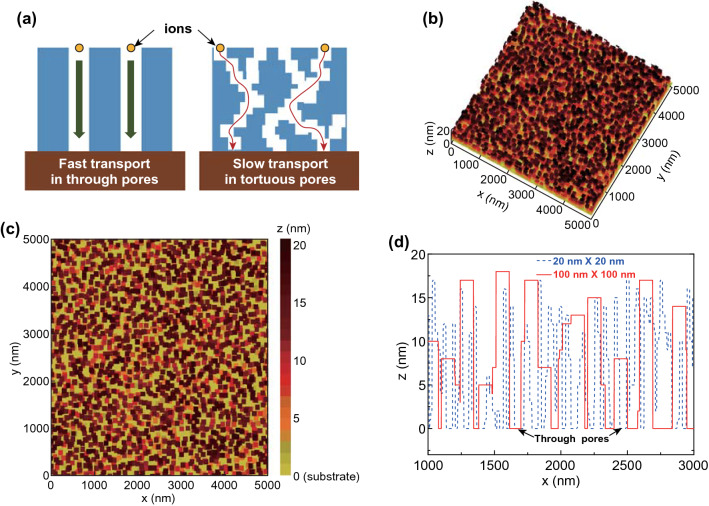
Simulation of self-assembly of passivated graphene (2D flakes) into 3D morphology with macroscale through pores. **a** Schematic illustration of different ion transport in through pores and tortuous pores. **b**, **c** Simulation results of the 3D gas lattice model of Rabani et al. for the final morphology after drying of thin-film liquid solutions containing graphene flakes (Supplementary Videos S1 and S2 for the evolution of the porous structures). **d** Height profile of the simulated morphology for graphene flakes of two different lateral sizes. Both indicates the occurrence of through pores

The dynamics of the model are stochastic. Both the liquid fluctuation and micro-flake diffusion processes take place with a Metropolis probability given by Eq. 1:

1 $$p_{\text{acc}} = { \hbox{min} }\left[ {1,{ \exp }\left( { - \Delta H/k_{\text{B}} T} \right)} \right]$$

where *k*_B_ is the Boltzmann constant, *T* is the temperature, and Δ*H* is the change in energy resulting from the process (liquid fluctuation or micro-flake diffusion) with the Hamiltonian *H* being calculated as Eq. 2:

2 $$H = - \varepsilon_{l} \mathop \sum \limits_{ij} l_{i} l_{j} - \varepsilon_{n} \mathop \sum \limits_{ij} n_{i} n_{j} - \varepsilon_{nl} \mathop \sum \limits_{ij} n_{i} l_{j} - \varepsilon_{ns} \mathop \sum \limits_{ij} n_{i} s_{j} - \varepsilon_{ls} \mathop \sum \limits_{ij} l_{i} s_{j} - \mu \mathop \sum \limits_{i} l_{i}$$

where $\varepsilon_{l}$, $\varepsilon_{n}$, $\varepsilon_{nl}$, $\varepsilon_{ns}$, and $\varepsilon_{ls}$ are attraction strength between two liquid cells, two micro-flake cells, one micro-flake cell and one liquid cell, one micro-flake cell and one substrate cell, and one liquid cell and one substrate cell, respectively, and $\mu$ is chemical potential which is used to establish the average concentration of liquid and vapor cells at equilibrium. Each simulation step consists of an attempt to change the value (*l*_*i*_) of all the liquid/vapor cells followed by *N*_move_ attempts to move all the nanoparticles. For the simulations in Figs. 1b, c and S1, S2, the following parameters are used: $\varepsilon_{l} = 2k_{\text{B}} T$, $\varepsilon_{n} = 2\varepsilon_{l}$, $\varepsilon_{nl} = \varepsilon_{ns} = 1.5\varepsilon_{l}$, $\varepsilon_{ls} = \varepsilon_{l}$, $\mu = - 3.125\varepsilon_{l}$, and *N*_move_ = 20. The simulation box is 5 µm long, 5 µm wide, and 20 nm high with periodic boundary conditions in the horizontal directions and open boundary conditions in the vertical direction. The initial volume concentration of the micro-flakes is 25%, i.e., 25% of the cells (excluding the substrate cells) are occupied by micro-flakes.

### Synthesis of Passivated Graphene Micro-flakes

The passivated graphene micro-flakes were synthesized through sequent in situ reduction of potassium permanganate (KMnO_4_) and polymerization of aniline at the presence of graphene flakes. First, graphite foil was electrochemically exfoliated in dimethylformamide (DMF) to obtain graphene/DMF dispersion (~ 2 mg mL^−1^) [5], followed by bath sonication (at a power of 80 W) for 2 h, centrifugation at 10,000 rpm for 10 min and harvesting the supernatant. The XPS analysis [18] indicates the C/O ratio of the graphene is 7.8:1, while AFM analysis indicates the graphene mainly contains 3–6 flakes (Fig. S3). Second, KMnO_4_ aqueous solution (5 mL, 10 mg mL^−1^) was added dropwise to the graphene/DMF supernatant (20 mL), followed by adding ethanol (2.5 mL) dropwise and stirring for 5 h to reduce KMnO_4_ to manganese oxide (MnO_*x*_ or Mn_3_O_4_ as identified in Fig. S3) nanoparticle coating on the graphene flakes. After washing with deionized water and isopropanol, the graphene/MnO_x_ flakes were dispersed in propylene glycol (10 mL). Third, graphene/MnO_x_/propylene glycol dispersion (4 mL) was mixed with dipropylene glycol butyl ether (20 mL), aniline (96 µL), and concentrated hydrochloric acid (88 µL, 38%) under stirring, followed by adding dropwise ammonium persulfate aqueous solution (1.6 mL, 14 wt%) and stirring for 5 h to polymerize the aniline and obtain doped passivated graphene flakes (hereafter denoted as GMP flakes). After washing with water and isopropanol, the doped GMP flakes were dispersed in propylene glycol (8 mL) to obtain green dispersion. Finally, ammonium solution (224 µL, 28%) was added to the doped GMP/propylene glycol dispersion and stirred for 1 h to de-dope the GMP flakes. After washing, the de-doped GMP flakes were dispersed in 8 mL ethylene glycol to obtain stable dark-blue dispersion. Using a high-precision balance (Advanced Analytic balance with precision of 0.01 mg, METTLER TOLEDO), the GMP concentration is estimated to be about 2.6–3.3 mg mL^−1^.

### Ink Preparation

The GMP/ethylene glycol dispersion was directly used as inks for inkjet printing. The poly (3,4-ethylenedioxythiophene)-poly(styrenesulfonate) (PEDOT:PSS) ink was formulated through mixing commercial PEDOT:PSS aqueous solution (1.1 wt%, Product No. 739332, Sigma-Aldrich) with deionized water and diethylene glycol butyl ether at the volume ratio of 1.0:2.6:0.4. The PVA/H_2_SO_4_ gel electrolyte was prepared through mixing PVA (200 mg), fumed SiO_2_ nanoparticles (7 mg, Product No. S5130, Sigma-Aldrich), and H_2_SO_4_ (600 mg) with deionized water (2.5 mL) and stirring at 90 °C until the dispersion was clean. The PVA/H_2_SO_4_ electrolyte ink was prepared through mixing PVA/H_2_SO_4_ gels (0.3 mL) with deionized water (5.1 mL) and ethylene glycol (0.6 mL), followed by stirring and centrifugation to remove the big particles. The gold inks for printing of current collectors and micro-pillars are commercial gold nanoink (UTDAu40, UtDots Inc.) and Au nanometal ink (ULVAC Inc, Japan), respectively.

### Ink Printing (Device Fabrication)

Except the gold micro-pillars, all the components of the micro-supercapacitors were printed with the Dimatix Materials Printer (DMP 2800, FujiFilm Inc.) equipped with 10 pL cartridges (DMC-11610). Table S1 lists the key parameters for the inkjet printing via the DMP printer for different inks. The areal porosity of the printed patterns was measured through analyzing the optical micrograph with the Gwyddion software. The gold micro-pillars were printed with a commercially available electrohydrodynamic inkjet printer, Super-fine inkjet printer (SIJ Technology Inc., Japan). During printing, ink droplets were pulled out of the nozzle by a high-strength electric field between the ink meniscus and the substrate [19]. The volume of the ejected droplets was optimized by adjusting the bias and peak-to-peak voltage, the distance between the nozzle tip and the substrate, and the ejection frequency. Layer-by-layer approach was used to build up the micro-pillars. The width and height of the micro-pillars were controlled by adjusting the volume of ink deposited in each layer, the overall number of layers, and the time delay between printing the subsequent layers (to facilitate solvent evaporation).

### Electrochemical/Electrical Characterization

All the electrochemical measurements (cyclic voltammetry, galvanostatic charge–discharge, and electrochemical impedance spectroscopy) and the electrical measurements for the PEDOT:PSS conductance were performed at two-terminal configuration with the use of a Gamry Interface 1010E potentiostat (Gamry Instruments Inc., Warminster PA, USA), which was connected with a Signatone S-1160 probe station equipped with S-725-PRM micropositioners (Signatone Corporation, Gilroy CA, USA).

The areal capacitance extracted from cyclic voltammetry was obtained from Eq. 3:

3 $$C_{A} = \frac{{\mathop \smallint \nolimits_{0}^{\Delta V} \left( {I_{\text{C}} - I_{\text{D}} } \right){\text{d}}V}}{2Av\Delta V}$$

where *I*_C_ and *I*_D_ are the charging and discharging currents, respectively, $\Delta V$ is the voltage window (= 1 V), $v$ is the scan rate, and $A$ is the footprint area (~ 0.21 cm^2^) of the micro-supercapacitors including the finger gaps.

The areal energy density (*E*_A_) and power density (*P*_A_) in the Ragone plot are calculated from Eqs. 4 and 5:

4 $$E_{\text{A}} = \frac{1}{2} \cdot C_{\text{A}} \Delta V^{2}$$

and

5 $$P_{\text{A}} = \frac{{E_{\text{A}} }}{t} = \frac{{E_{\text{A}} v}}{\Delta V}$$

where $t = \Delta V/v$ is the discharging time.

## Results and Discussion

To improve the compactness of electronic systems, MSCs are expected to have miniaturized footprint area (lateral dimension) whereas the electrode thickness (vertical dimension) could be as large as possible (provided that the thickness does not negatively impact the micro-fabrication process) [1] in order to maximize the stored energy. Notwithstanding, the mere fabrication of thick electrodes is not sufficient for supercapacitors to attain high rate capability. The increase in electrode thickness often makes the ion transport paths lengthy and complex (as illustrated in Fig. 1a) and hence impedes the performance at high charge–discharge rates (high scan rate or large current density) [20]. Recently, many studies have demonstrated that hierarchical porosity with multiscale pores, especially macroscale (µm-level) pores [21, 22] and open/straight pores with less tortuosity [23, 24], can effectively improve ion diffusion (Fig. 1a) during the fast charging/discharging processes. However, usually the fabrication of such hierarchically porous structures requires complicated material engineering or fabrication processes, and the high performance must rely on liquid-state electrolytes which have high ionic conductivity but are not preferred for micro-supercapacitor fabrication due to the increased difficulty of encapsulation [1].

### Simulation

In this work, we have developed an inkjet printing process to produce thick electrodes with 3D networked microstructure which contains macroscale open pores. The process is based on the drying-mediated self-assembly behavior of 2D materials. Over the past two decades, it has been well established that during the evaporation of thin-film solutions containing passivated nanoparticles, the nanoparticles can self-assemble (aggregate) into various interesting patterns or structures, such as wormlike [17], labyrinthine [25], fingering [26], and cellular [27] patterns. The general mechanism of such self-assembled patterns relies on the interaction between the nanoparticle aggregation and the solvent evaporation: the passivated nanoparticles are initially stable in the solvent, but start to aggregate with each other when the solvent evaporates. Because the nanoparticles have comparable size to the typical solvent correlation length (~ 1 nm), their movement (diffusion) and aggregation are strongly restricted by the evaporation of the thin-film solvent; and meanwhile, the nanoparticle movement also significantly influences the dynamics of the solvent evaporation. As a result, the mutual impacts generate a variety of intriguing patterns under different drying conditions. Based on this mechanism, Rabani et al. [17, 28] introduced a generic coarse-grained lattice gas model to simulate the self-assembly behavior of the nanoparticles and account for almost all observed spatial and temporal structures and patterns. As an important extension, we employ the 3D lattice gas model [28] to explore the self-assembly behavior of the emerging graphene (as well as other 2D materials). The extremely high anisotropicity of graphene flakes leads to distinct self-assembly behavior from spherical nanoparticles. Their atomic thickness (or nm-scale thickness for few-layer flakes) makes their aggregation still strongly impacted by the solvent fluctuations, whereas their µm-scale lateral dimension gives rise to significantly increased attractions with the solvent and other flakes that also influence the final pattern formation. A typical simulation result of the evolution of graphene self-assembly is shown in Supplementary Videos S1 and S2. Initially, the graphene flakes are uniformly distributed and can diffuse freely in the 3D simulation box. During the liquid evaporation, the flakes start to aggregate and are finally frozen to form a 3D network morphology (Fig. 1b, c). Interestingly, unlike other graphene-based 3D porous structures [29], the drying-mediated 3D graphene networks contain plenty of macroscale “through pores” which penetrate from the top to the bottom of the dried films and expose the substrate (Fig. 1c, d), even when the solution contains very high concentration of graphene (e.g., initially 25% volume concentration in the simulations for Fig. 1b, c). Consistent with the early studies [17, 28], the network structure is not sensitive to the size of nanoparticles (Fig. S1). However, increasing the flake size enlarges the through pores and pushes more graphene stacked upon each other to form higher graphene clusters (Figs. S1 and S2). The latter is because larger size increases the collision probability and inter-particle attractions, both of which enhance the formation of 3D structures [28] during solvent evaporation. Thereby, 2D materials of large flake size are favored for *simultaneously* producing two “special” morphology characteristics: thick 3D network structure, and large through pores. In contrast, for spherical/isotropic nanoparticles, it is challenging to build up thick/high 3D structures and meanwhile keep the large-size through pores [28, 30].

### Inkjet Printing

**Fig. 2:**
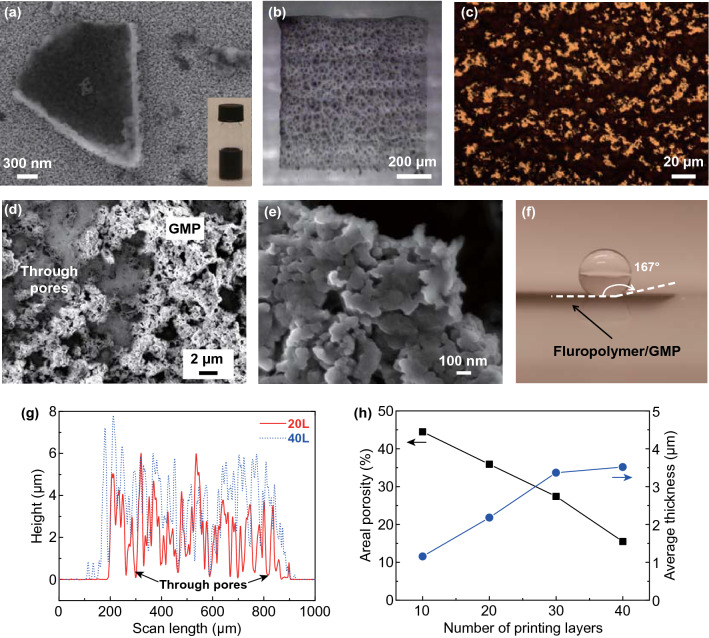
Inkjet-printed GMP patterns with through pores. **a** An SEM image of a printed large GMP flake (on top of printed gold nanoparticles). Inset: photograph of the GMP ink. **b** Micrograph of a drying GMP thin-film liquid pattern (Supplementary Video S3 for the whole drying process). **c** Micrograph of a fully dried GMP film (printed on top of a gold film). **d**, **e** SEM images of a dried GMP film (with 40 printing passes). **f** Photograph of a deionized water droplet on the surface of a printed GMP pattern coated with a fluoropolymer. **g** Height profile of dried GMP patterns printed with different passes. **h** Areal porosity and average thickness of printed GMP patterns against of the number of printing layers

Because of the simple processing, such an evaporation-driven self-assembly was envisioned as a promising “bottom-up” approach for novel device fabrication [16, 17], yet so far few has been demonstrated. Inkjet printing is an ideal technique to implement this approach for device fabrication because of its ability to manipulate tiny droplets at pico-liter or even femto-liter level to deposit liquid-phase inks in arbitrary thin-film patterns on various substrates. In this work, we use inkjet printing to readily produce graphene films/patterns with the self-assembled 3D networked microstructure. In general, the drying-mediated self-assembly requires that the nanoparticles have relatively weak inter-particle attractions, which are efficiently screened in a solution (to avoid agglomeration), but become manifest as the solvent evaporates [17]. Although a number of studies formulate various inkjet printable inks for graphene and 2D materials, similar microstructures to that in Fig. 1b, c have not been obtained in a controllable manner. Many of the present inks use binders (or surfactants) [13, 31] or aggressive solvents, such as *N*-methyl-2-pyrrolidone (NMP) [32, 33], to stabilize graphene, which, however, also prevent the self-assembly of graphene during the solvent evaporation. This often results in non-porous films/patterns. Here we prepare passivated graphene by sequent in situ synthesis of two different nanoparticles: manganese oxide nanoparticles are first synthesized through in situ reduction of KMnO_4_ at the presence of the electrochemically exfoliated *pristine* graphene flakes [34] to obtain manganese oxide-anchored graphene flakes (Fig. S3), which are further passivated through in situ polymerization of aniline to form polyaniline (PANI)-anchored graphene flakes (Figs. 2a and S3), or the so-called GMP micro-flakes. Assembly of nanomaterials onto graphene flakes (usually graphene oxide flakes) has become a popular technique in developing new devices and nanocomposite materials [35] because it can make the best of the macromolecular structure and excellent properties of graphene to improve performance or produce more functionalities. As a new successful case, the GMP micro-flakes developed in this work enable us to formulate stable inks simply through dispersing them in a mild solvent, ethylene glycol. The high-concentration ink (about 3.0 mg mL^−1^, Fig. 2a) is stable for more than 1 year and provides excellent jetting performance (Fig. S4). It is worth noting that the dual-passivation process plays an important in the ink stability because neither the *singly* manganese oxide-passivated or PANI-passivated graphene can attain comparable stability in the inks. The accurate mechanism needs further exploration. One possible reason is that the synthesis process of manganese oxide subtly modifies the graphene flakes so as to improve the coverage of anchored PANI while still retains their good conductivity. In particular, during the ink drying, the GMP flakes self-assembled into a 3D network structure (Fig. 2b) which is qualitatively similar to the simulated morphology in Fig. 1b, c, i.e., containing plenty of macroscale through pores (Fig. 2c, d, g). The through pores remain even when the networks are as thick as several micrometers (Fig. 2g, h). It is worthy of mentioning that the size of the through pore in the printed GMP patterns is at the level of 10 microns, much larger than that in the simulated patterns (Figs. 1c and S1). The deviation might result from the different factors between simulations and experiments, such as the thickness of the thin-film liquids, graphene flake size (about 0.5–5 µm for the experimental GMP flakes, as shown in Figs. 2e and S3c), and motions of graphene flakes during aggregation (see Sect. 2 for details). Nevertheless, the large-scale through pores play an important role in the inkjet printing of high-rate MSCs, as demonstrated below.

In general, inkjet printing is only suitable for printing thin-film patterns (< 1 micron) when high resolution is required. Although the high alignment accuracy allows for overwriting for multiple passes to increase the film thickness, the patterns formed in the initial printing passes are often damaged or deformed by the ink solvents from the subsequent printing passes, which causes short circuits of the interdigitated electrodes in the case of MSCs and hence limits the electrode thickness [5]. Nevertheless, the initially printed porous GMP patterns can act as “sponge” to quickly absorb the subsequent inks and restrict them within the predefined area. During drying of the absorbed inks, the GMP flakes prefer to aggregate onto the underlying GMP clusters so as to increase the pattern thickness while retain the 3D network morphology, which can further absorb inks from the following printing passes. Such a self-replication process makes it possible to overwrite the patterns for hundreds of printing passes without causing evident deformation (Fig. S5). In addition, as indicated in Fig. 2g, although the printed GMP patterns are very porous, there is no evident thickness difference between the perimeter and center of the patterns. In other words, the GMP patterns are free from the ubiquitous coffee-ring effect [36]. In general, during the drying of a liquid drop, outward capillary flows often occur due to the pinning of the three-phase contact line. They carry solutes from the center to the perimeter and make the latter much thicker, resulting in the so-called coffee-ring effect. In this work, the underlying 3D porous structures absorb and localize the inks and hence prevent the global outward flows, as evidenced by the factor that in comparison with the ink drying in the first printing pass (Supplementary Video S3), the global flows attenuate in the subsequent printing passes (Supplementary Videos S4 and S5). The absence of global outward flows essentially suppresses the coffee-ring effect. Additionally, the multiscale porous microstructure (Fig. 2d, e) of the GMP patterns significantly increases the surface roughness of the substrates and thereby can geometrically enhance the hydrophobicity of a hydrophobic solid [37] to form a super-hydrophobic surface (water contact angle > 150°). As shown in Figs. 2f and S6, after dip coating or drop casting of a thin layer of fluoropolymer, the GMP patterns attain a water contact angle around 170°, much higher than 109° for the fluoropolymer coating on a flat surface (Fig. S6). Usually the production of super-hydrophobic surface needs expensive materials and complex time-consuming processes [38]. In this sense, our inkjet printing of GMP provides a simple and cost-effective solution. On the whole, the global uniformity, large thickness, and multiscale porous microstructure are all favorable for high-performance MSCs.

### Printed High-Rate MSCs

**Fig. 3:**
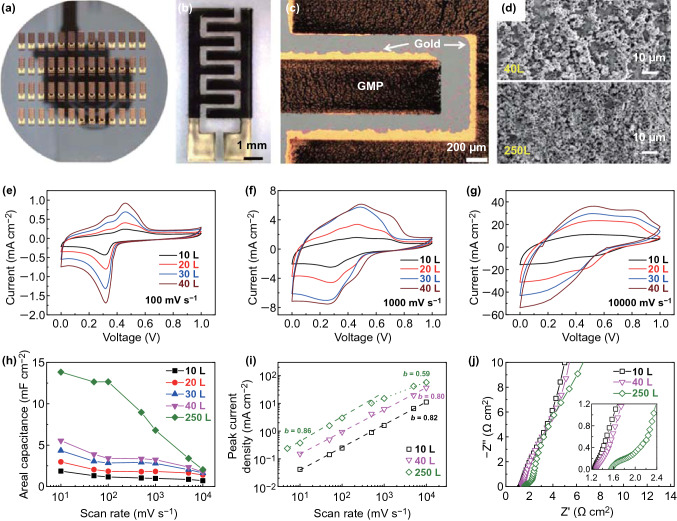
Inkjet-printed GMP MSCs with printed gold current collectors and GMP electrodes. **a** Photograph of printed MSCs on a 4-inch Si wafer. **b**, **c** Micrographs of a printed device (40 GMP layers) on glass. **d** SEM images of GMP patterns printed with 40 (upper) and 250 (lower) layers. **e**–**g** CV of the printed MSCs at different scan rates. **h** Dependence of the areal capacitance on scan rates for MSCs with different GMP layers. **i** Power-law fitting of the dependence of peak current density (in charging part of the CV curves) on scan rate. **j** Impedance spectra for the devices with different GMP layers. The inset is the zoom-in view of the high-frequency region

Thus, MSCs with GMP electrodes can be easily printed on various substrates (Fig. 3a, b). First, gold nanoparticles are printed in interdigitated structures (with gap of ~ 200 µm) to serve as current collectors, then the GMP flakes are printed at various printing layers (passes) as porous electrodes, and finally, a hydrogel electrolyte (aqueous dispersion of polyvinyl alcohol (PVA), sulfuric acid, and silica nanoparticles) is drop-cast on the top to bridge the two separate electrodes. Due to the self-replication behavior of the micro-flake inks, the electrodes are well patterned and free from short circuits (Fig. 3b, c). It is worth noting that the through pores, which remain in the 40-layer devices, seem to vanish in the 250-layer devices (Fig. 3d). One possible reason is that when the porous patterns become very thick (e.g., over 15 µm thick for the 250-layer devices, Fig. S7), most of the subsequently printed ink is trapped into the through pores, which restricts the motion of the GMP flakes inside the through pores and forces them to be filled after ink drying. Nevertheless, the inks on the top of the patterns still have freedom to assemble GMP flakes and continue the self-replication process, as evidenced by the presence of deep (~ 5 µm thick) pores at the top of the 250-layer devices (Fig. S7). As shown in Fig. 3e-g, the cyclic voltammograms (CVs) contain characteristic pseudocapacitive peaks [39] of PANI which shift with the increase of scan rates. In the literature, supercapacitors based on PANI, graphene/PANI [39–41] or other pseudocapacitive materials [42] are typically restricted to a moderate scan rate of up to 100 mV s^−1^, while our printed GMP MSCs (with < 40 printing layers of GMP) exhibit good rate capability (Fig. 3e–g) and vertical scalability against printing passes (or thickness, Fig. 3h) at high scan rates up to 10 V s^−1^. To confirm the high-rate performance, we analyze in Fig. 3i the dependence of peak current *i*_p_ on the scan rates *v*, in terms of the assumed power law of *i*_p_ = *av*^*b*^ where *a* and *b* are pre-factor and exponent, respectively. In general, *b* ≈ 1 indicates a high-rate capacitive storage mechanism while *b* ≈ 0.5 corresponds to a slow storage process limited by diffusion [20, 24]. For all of our GMP MSCs with less than 40 printing passes, *b* ≈ 0.8 throughout the scan rates from 10 to 10,000 mV s^−1^, suggesting the fast capacitive storage mechanism. It should be ascribed to the presence of the through pores which are of macroscale and decreased pore tortuosity, two favorable characteristics to shorten the ionic transport lengths [24]. As compared with the macropores (typically less than 1 μm) in other studies [21, 24], the through pores in our GMP devices have much larger size (at the level of 10 μm). Although the large pore size lowers the mass loading of active materials, it is likely an enabling factor for solid-state electrolytes (of low ionic conductivity yet preferred for on-chip MSCs due to simplified encapsulation) [1] to achieve high-rate capability. On the contrary, due to the absence of through pores, the thicker micro-supercapacitor (250 printing layers) has inferior rate performance (Fig. 3h). While it has an areal capacitance of about 13.8 mF cm^−2^ at low scan rate of 10 mV s^−1^, when the scan rate increases to 10 V s^−1^, its capacitance reduces to 2.0 mF cm^−2^, very close to that (1.7 mF cm^−2^) of the 40-layer device. In the *i*_p_ ~ *v* plot (Fig. 3i), *b* for the 250-layer device keeps high value of 0.86 only at low scan rates up to 100 mV/s and decreases to 0.59 at high scan rates of 500–10,000 mV s^−1^, implying the slow diffusion-limited storage process. This is consistent with the 45° Warburg-type impedance element at the mid-frequency region in the Nyquist plots for the 250-layer device (Fig. 3j) [20], clearly different from the nearly vertical curves of the thinner (10- and 40-layer) devices. To further confirm the significance of the through pores, we have also formulated pure PANI inks by dispersing commercial PANI in dimethyl sulfoxide (DMSO) at a concentration of 3 mg mL^−1^ and printed MSCs in the same device structure as the GMP devices. Also, because of the lack of through pores (Fig. S8), the devices exhibit poor performance at high scan rates despite the comparable capacitance to that of the GMP devices at low scan rates.

### Heterogeneous 3D MSCs

**Fig. 4:**
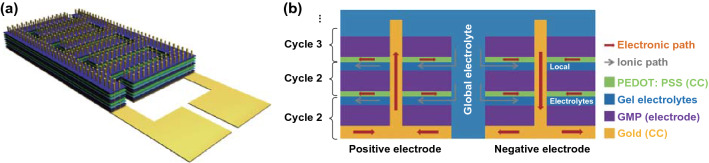
Schematics of the structure of the 3D heterogeneous MSCs. **a** 3D view and **b** cross section

**Fig. 5:**
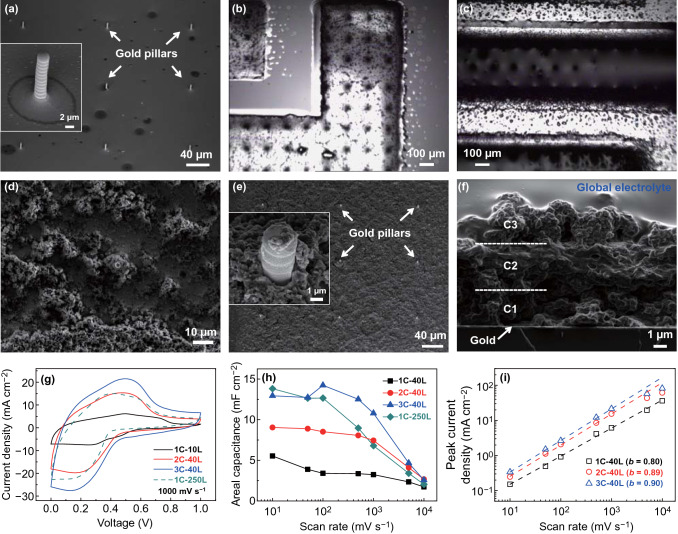
Fully inkjet-printed 3D structured GMP MSCs. **a** SEM image of the printed 3D current collectors. Inset: close-up view of a gold pillar. Micrographs of printed (**b**) GMP inks and (**c**) PVA/H_2_SO_4_ gel inks (before drying) on the 3D current collectors. **d** SEM image of printed 40-layer GMP surrounding a gold micro-pillar. SEM images of top view (**e**) and cross-sectional view (**f**) of the final 3-cycle devices. **g** CV of the 3D devices with different cycles at a scan rate of 1000 mV s^−1^. **h** Dependence of the areal capacitance on scan rates for devices with different cycles. **i** Power-law fitting of the dependence of peak current density (in charging part of the CV curves) on scan rates

Therefore, the high-rate performance of the inkjet-printed GMP MSCs is restricted to the electrode thickness of a few micrometers. In order to retain the high-rate performance for thicker electrodes, we have fabricated through full printing 3D structured MSCs (Fig. 4a, b) whose electrodes consist of cyclic stacking of current collectors, electrodes, and gel electrolytes. In striking distinction from all the other supercapacitor electrodes in the literature, multiple continuous electrolyte layers are embedded in the 3D electrodes to maximize their interface area and provide faster ion transport pathways. However, electrolytes are electronically insulating which will reduce the overall electrode conductivity and degrade the rate capability. To circumvent this drawback, we introduce in each electrode a sparse array of metal micro-pillars to connect the upper current collectors with the base current collectors and form conductive electronic paths, so that multiple ionic and electronic pathways are constructed throughout the 3D electrodes (Fig. 4a, b). More importantly, such 3D MSCs can be readily fabricated by inkjet printing. On top of the inkjet-printed base gold current collectors, we use high-resolution electrohydrodynamic inkjet printing [43, 44] to print gold pillar arrays where the pillars have diameter and height about 2 and 10 µm, respectively, and are pitched by 100 µm (Fig. 5a, Supplementary Video S6, Sect. 2). Then the GMP inks are printed for 40 layers to fabricate the multiscale porous electrodes (Fig. 5b, d, Supplementary Video S7), followed by printing of 10 layers of PVA/H_2_SO_4_ aqueous inks for the local electrolyte layers (Figs. 5c and S9a–c). These constitute the first cycle. Each successive (upper) cycle starts with printing of 40 layers of PEDOT:PSS inks as the upper (local) current collectors (Fig. S9d–f), followed by the printing of GMP and PVA/H_2_SO_4_ layers with the same process as in the first cycle. Finally, viscose PVA/H_2_SO_4_ gels are drop-cast as the global electrolyte to bridge the pair of 3D electrodes. In this work, we have managed to fabricate devices with three cycles (Fig. 5e) where the topmost GMP layer has approached the top of the gold pillars, suggesting a total electrode thickness about 10 µm (Figs. 5f and S9g–i). In Fig. 5, a 3D device is denoted as “*m*C-*n*L” which means the device comprises *m* cycles and each cycle contains *n* layers of GMP. The CV analysis (Fig. 5g, h) confirms the excellent vertical scalability (areal capacitance against printing cycles or thickness) under scan rates up to 1 V s^−1^. The 3-cycle MSC (in total of 120 printing layers of GMP) has attained areal capacitance of 11 mF cm^−2^ at 1 V s^−1^, much higher than 7 mF cm^−2^ for 1-cycle device with 250 layers of GMP. The *i*_p_-*v* curves of the 2- and 3-cycle devices (Fig. 5i) retain a high value of *b* ≈ 0.9 at scan rates up to 1 V s^−1^, confirming the high-rate capacitive storage process. At scan rates > 1 V s^−1^, the 2- and 3-cycle devices exhibit similar capacitance, around 5 mF cm^−12^ at 5 V s^−1^ and 3 mF cm^−2^ at 10 V s^−1^. The degraded rate capability might result from the increased series resistance (Fig. S10) caused by the relatively low conductivity of PEDOT:PSS as the local current collectors. Nevertheless, the attained capacitance (> 10 mF cm^−2^ at 1 V s^−1^) has already been much higher than that of most other printed MSCs at much lower scan rates (< 2 mF cm^−2^ at 1–100 mV s^−1^) [45]. Additionally, the 3D devices exhibit good cycling performance with capacitance retention of 80% after being tested through galvanostatic cycling for 2000 times at high charging/discharging current density of 2.5 mA cm^−2^ (Fig. S11).

The printing of 3D MSCs makes use of the synergy between different printing layers. The porous GMP layers can quickly absorb the subsequently printed GMP and PVA/H_2_SO_4_ inks so as to retain the interdigitated structure. The sulfuric acid in the electrolyte inks can directly re-dope the underneath GMP layer (PANI on the GMP flakes is initially de-doped; see Sect. 2 for details) to increase its electrical conductivity. The aqueous PEDOT:PSS inks dry almost immediately after being printed on the substrates so that they do not impact the underlying PVA/H_2_SO_4_ electrolyte layers. Instead, after drying the cross-linked PEDOT:PSS layers can serve as both the conductive current collectors and the protective layers for the PVA/H_2_SO_4_ layers to prevent them from being attacked by the upper GMP inks. Besides, the ethylene glycol in the GMP inks serves as an effective dopant for the underlying PEDOT:PSS layer to greatly improve its conductance [46] (Fig. S12). Finally, all the three different material layers can attain the functionality immediately after ink drying, *i.e.*, in no need of any post treatment such as annealing and chemical reduction—the pristine graphene in the GMP flakes avoids any reduction process after printing. All these merits enable us to directly print a number of heterogeneous layers for hundreds of passes without severely deforming the patterns (Fig. S13).

### Performance Comparison

**Fig. 6:**
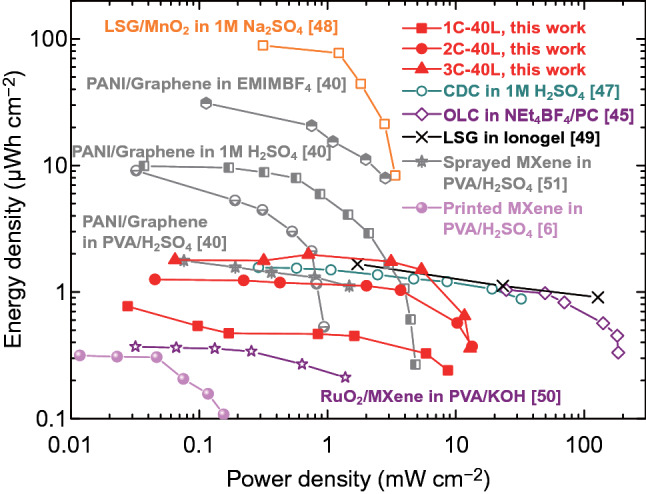
Areal-normalized Ragone plots showing the energy density versus power density of the 3D structured GMP MSCs. Also included are several interdigitated MSCs fabricated with other techniques and/or different electrode materials and electrolytes. *LSG* laser-scribed graphene, *CDC* carbide-derived carbon, *OLC* onions-like carbon. Numbers in the brackets are the cited references

Since areal performance is a key metric for on-chip MSCs [1, 47], we compare the areal energy density and power density of our printed devices with those of the advanced MSCs in the literature [6, 40, 45, 47–51] fabricated with various advanced electrode materials and/or fabrication techniques as shown in Fig. 6. It is clear that our printed devices have attained comparable performance to most of the other devices, with areal energy density of ~ 1 µWh cm^−2^ at power density of ~ 10 mW cm^−2^ for the 3-cycle devices. It is worth mentioning that many of the other MSCs are actually characterized with liquid electrolytes, which result in higher electrochemical performance, but significantly increase the difficulty of encapsulation. The simple processing of our fully printed all-solid-state MSCs greatly improves compactness and integrability on chip.

## Conclusions

In conclusion, we have formulated stable micro-flake inks of polyaniline and manganese oxide-passivated graphene nanocomposites that allow to directly print multiscale porous patterns with similar morphology to the simulation results. The porous structures can absorb the multiple printed layers of ink and prevent their local flows to enable facile fabrication of thick (over 10 µm) yet globally uniform patterns at high resolution around 100 µm. In particular, the presence of through pores greatly improves the macroporosity and decreases the pore tortuosity in the electrodes, leading to good rate capability of the all-solid-state MSCs at scan rates up to 10 V s^−1^. Moreover, likely for the first time, we have demonstrated fully inkjet-printed MSCs with innovative 3D electrode architecture that effectively integrates multiple cycles of current collectors, electrodes, and gel electrolytes with gold micro-pillars to achieve high-rate performance (> 10 mF cm^−2^ at scan rate of 1 V s^−1^) and excellent vertical scalability. The fully printed all-solid-state devices have attained comparable areal energy density and power density to most of other high-performance MSCs that are with liquid-state electrolytes and fabricated through more complicated processes. The discovered self-assembly behavior of graphene flakes, the developed inkjet printing techniques, and the demonstrated 3D electrode architecture can all be generalized through integrating with other materials (especially other pseudocapacitive materials for passivation coating of graphene or other 2D materials) to provide simple yet scalable fabrication of high-rate MSCs to expedite on-chip integration of energy storage components in practice.

## Electronic supplementary material

Below is the link to the electronic supplementary material.

Supplementary material 1 (MP4 3224 kb)

Supplementary material 2 (MP4 5827 kb)

Supplementary material 3 (MP4 6710 kb)

Supplementary material 4 (MP4 9537 kb)

Supplementary material 5 (MP4 9869 kb)

Supplementary material 6 (MP4 9195 kb)

Supplementary material 7 (MP4 6527 kb)

Supplementary material 8 (PDF 2048 kb)
