# Supplementary material for: Drying-Mediated Self-Assembly of Graphene for Inkjet Printing of High-Rate Micro-supercapacitors
Source: Nanomicro Lett. 2020 Jan 27;12:40. doi: 10.1007/s40820-020-0368-8 (PMC7770958; doi:10.1007/s40820-020-0368-8)
Supplement: Supplementary file 8 — Supplementary material 8 (PDF 2048 kb) [file 40820_2020_368_MOESM8_ESM.pdf]

Supporting Information for

Drying-Mediated Self-Assembly of Graphene for Inkjet Printing of High-Rate Microsupercapacitors

Szymon Sollami Delekta[1], Mika-Matti Laurila[2], Matti Mäntysalo[2, *], Jiantong Li[1, 2, *]

[1]KTH Royal Institute of Technology, School of Electrical Engineering and Computer Science, Electrum 229, 16440 Kista, Sweden

[2]Tampere University, Faculty of Information Technology and Communication Sciences, Laboratory for Future Electronics, 33720 Tampere, Finland

*Corresponding authors. E-mail: matti.mantysalo@tuni.fi (Matti Mäntysalo); jiantong@kth.se (Jiantong Li)

S1 Description of the Supplementary Vidoes

Video S1 Simulation results (top view) of the 3D gas lattice model of Rabani *et al.* for the drying process of a thin-film (20 nm thick) liquid solution containing graphene flakes (each has lateral size of 100 × 100 nm^2 and thickness of 1 nm). The modeling and parameters are described in the Methods section. Only graphene flakes are shown in the video (i.e., solvents are not shown). The colors of the graphene flakes indicate their height position, with the same indicator as in Fig. 1c.

Video S2 3D view of the same simulation results as in Video S1

Video S3 Drying process of the 1st printing pass for an inkjet printed GMP square (800 µm × 800 µm) on glass slide. In the early stage of the drying process, global outwords flows occurred which could cause the "coffee-ring" effect.

Video S4 Drying process of the 3rd printing pass for the same GMP square as in Video S3. Throughout the drying process, no global outwords flows were observed though some local flows occurred

Video S5 Drying process of the 6th printing pass for the same GMP square as in Video S3

Video S6 Inkjet printing of gold micropillars on the base gold current collectors of the 3D-structured GMP microsupercapacitors through a super-fine inkjet printer. The golden region is the pizeroelectric inkjet printed gold base current collectors while the brown region is the SiO_2/Si substrate

Video S7 Drying process of the 1st printing pass of GMP electrodes on the 3D-structured gold current collectors (containing sparse arrays of gold micropillars)

S2 Supplementary Figures and Table

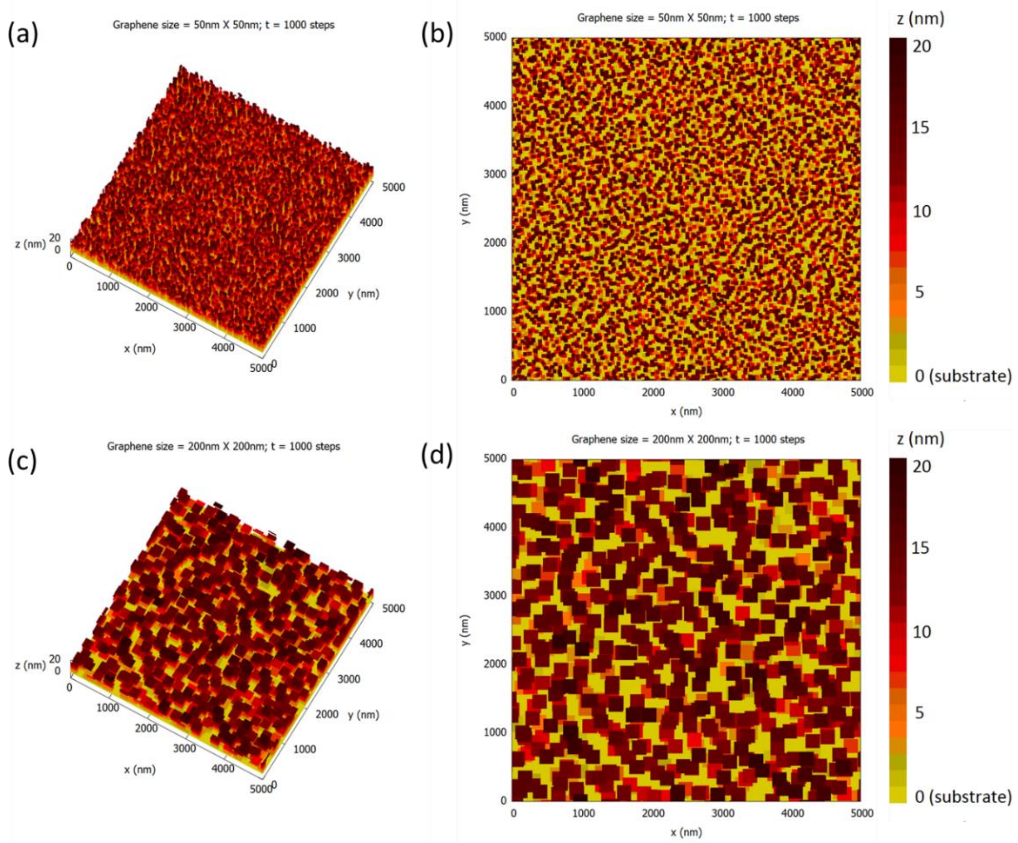

Fig. S1 Simulation results of the 3D gas lattice model of Rabani *et al.* for the dried patterns of thin-film liquid solution containing passivated graphene flakes. All the conditions are the same as in Fig. 1b, c expect that the lateral size of graphene flakes are 50 × 50 nm^2 in (**a, b**) and 200 × 200 nm^2 in (**c, d**)

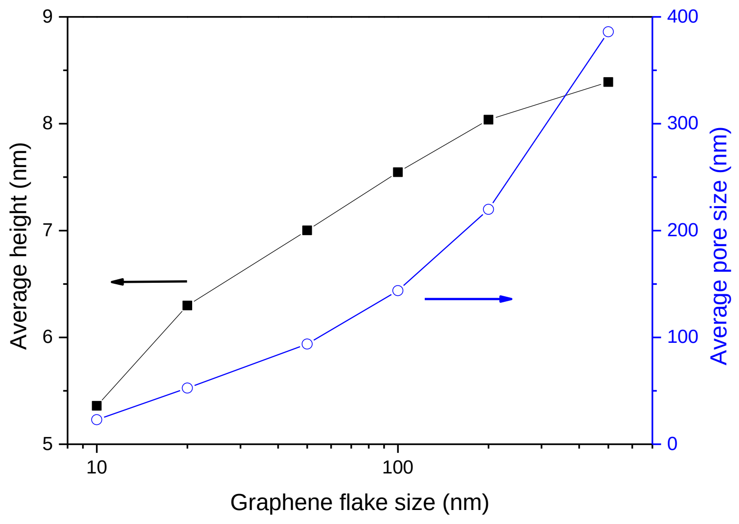

Fig. S2 Simulation results for the effects of the graphene flake size on the average height of all the graphene flakes in the final porous morphology (after 1000 Monto Carlo steps) and the average size (equivalent square length) of the through pores

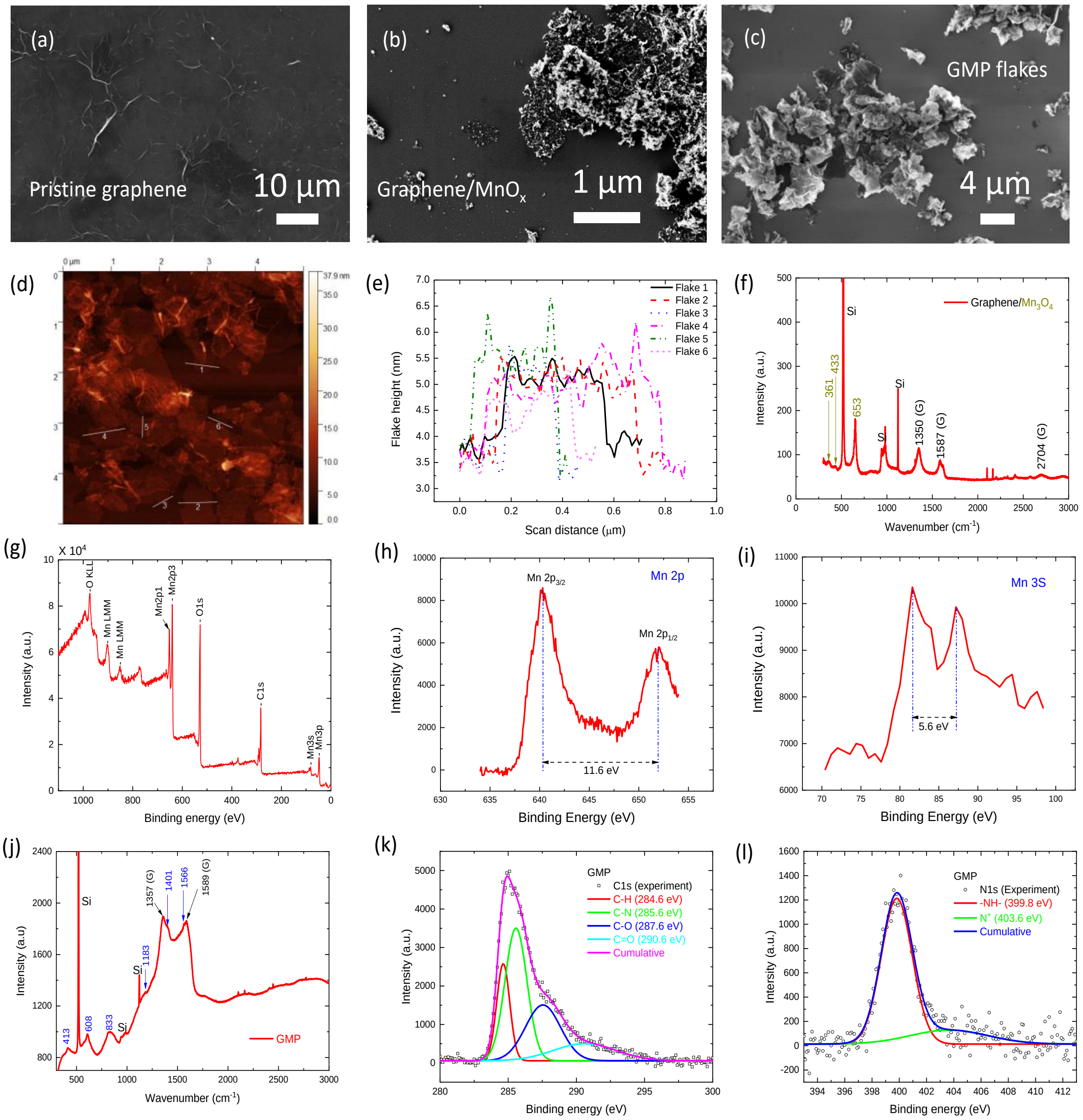

Fig. S3 Characterization of the prinstine graphene flakes, graphene/manganese oxide flakes and GMP flakes. (**a-c**) SEM images of (**a**) pristine graphene fabricated through electrochemical exfoliation (the SEM sample is a dried drop of graphene-DMF dispersion), (**b**) MnO_x-anchored graphene flakes (the SEM sample is a dried drop of MnO_x/graphene-propylene glycol dispersion) and (**c**) GMP micro-flakes (the SEM sample is a dried drop of GMP-ethylene glycol dispersion). (**d**) AFM image of prinstine graphene. (**e**) Height profile of graphene flakes marked in (**d**). (**f**) Raman and (**g-i**) XPS spectra of the graphene/manganese oxide composite. The manganese oxide could be identified as Mn_3O_4 in terms of the characteristic peaks at 361, 433, and 653 cm^{-1} in the Raman spectrum (**f**), the energy peaks at 640.8 eV and 652.0 eV of Mn 2p XPS spectrum (**h**), and separation of 5.6 eV in energy peaks of the Mn 3s XPS spectrum (**i**). (**j**) Raman and (**k**) XPS C 1s and (**l**) XPS N 1s spectra of the GMP composite. In (**k, l**), all the experimental binding energy was shifted by 2.0 eV to align the C–H and –NH– peaks to the literature values. The characteristic peaks (marked in blue) in (**j**) and the occurrence of C–N and –NH– peaks in (**k, l**) imply the formation of PANI onto the graphene flakes

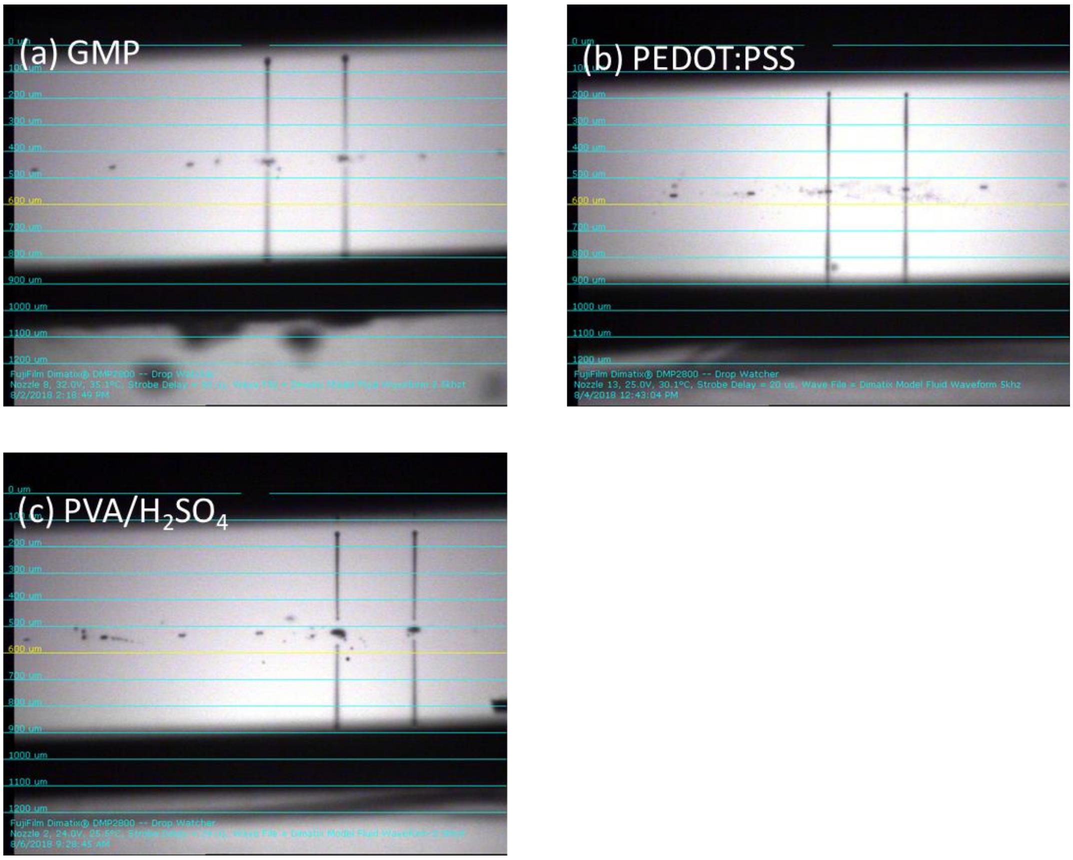

Fig. S4 Stroboscopic images of jetted drops indicating the jetting performance of the GMP inks (**a**), PEDOT:PSS inks (**b**) and PVA/H_2SO_4 inks. The images were captured using the Drop Watcher system of the Dimatix (DMP 2800) printer in the frozen mode with a strobe delay of 30 μs. To improve the printing reliability, during the printing of all the patterns and devices in this work, typically only 2 nozzles were used with the parameters in Table S1.

Table S1 Parameters for inkjet printing of various inks through the Dimatix printer

Inks	Drop spacing (μm)	Substrate temp. (°C)	Nozzle temp. (°C)	Interlayer delay (second)	Sintering
Gold (current collectors)	40	30	40	120	300 °C for 2 h
GMP	30	45	35	600	No
PVA/H_2SO_4	30	Room temp.	Room temp.	0	No
PEDOT:PSS	30	45	30	120	No

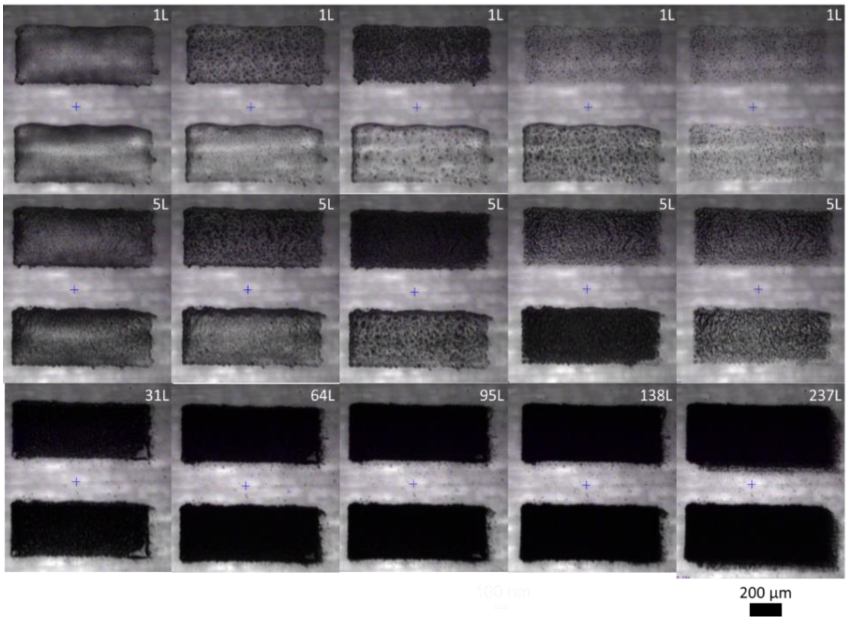

Fig. S5 Evolution of inkjet printing of a pair of small rectangular patterns (interspaced by 250 µm) with 237 printing passes (layers). The first and second rows show the as-printed and drying process of the patterns at 1st and 5th printing passes, respectively. The third row shows the dried patterns at more printing passes. It is clear even after being overwritten for about 250 printing passes, the geometry and dimension of the patterns can still remain unaltered, especially the gap distance. All the images share the same scale bar as in bottom-right corner.

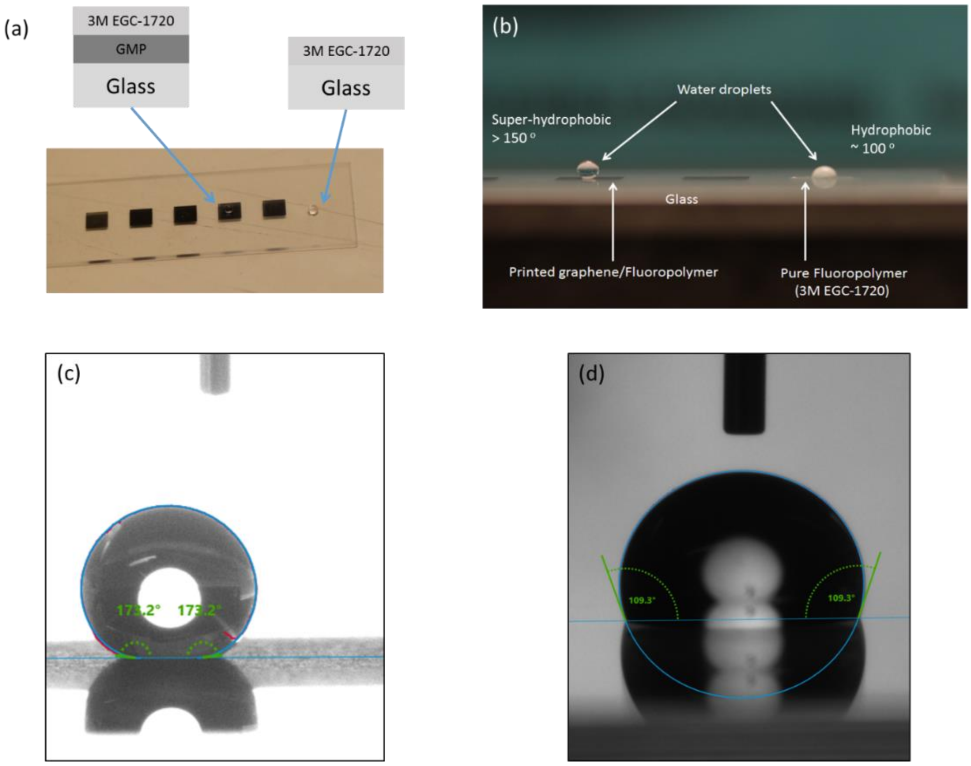

Fig. S6 Characterization of water contact angles on printed GMP patterns with fluoropolymer (3M EGC-1720) coating. Photographs in top view (**a**) and side view (**b**) of two water droplets of fluoropolymer coating on a glass slide (right) and printed GMP pattern (left). Static contact-angle images for the surfaces of fluoropolymer-coated GMP (**c**) and fluoropolymer-coated glass (**d**)

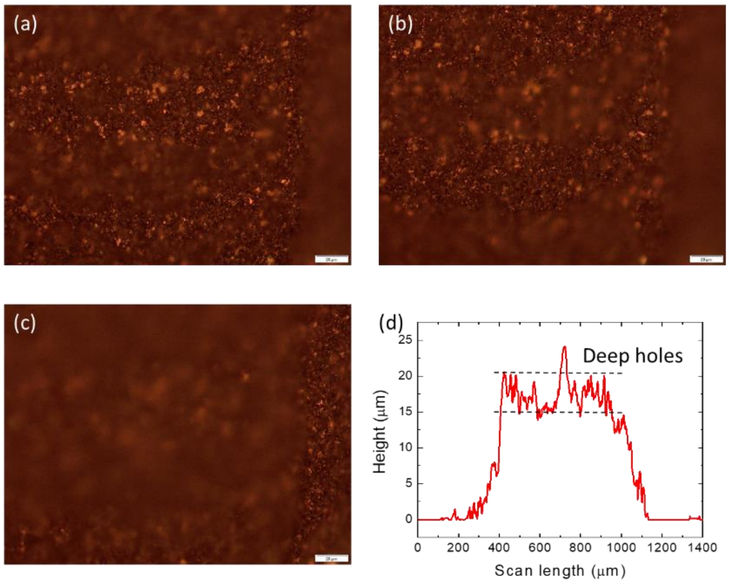

Fig. S7 Characterization of deep pores in the top of 250-layer GMP patterns. (**a-c**) Micrographs (top view) of the same position in the GMP patterns at different focus depth. The non-synchronous blurring regions indicating the presence of deep pores. Scale bars: 20 µm. (**d**) Height profile of a 250-layer GMP pattern

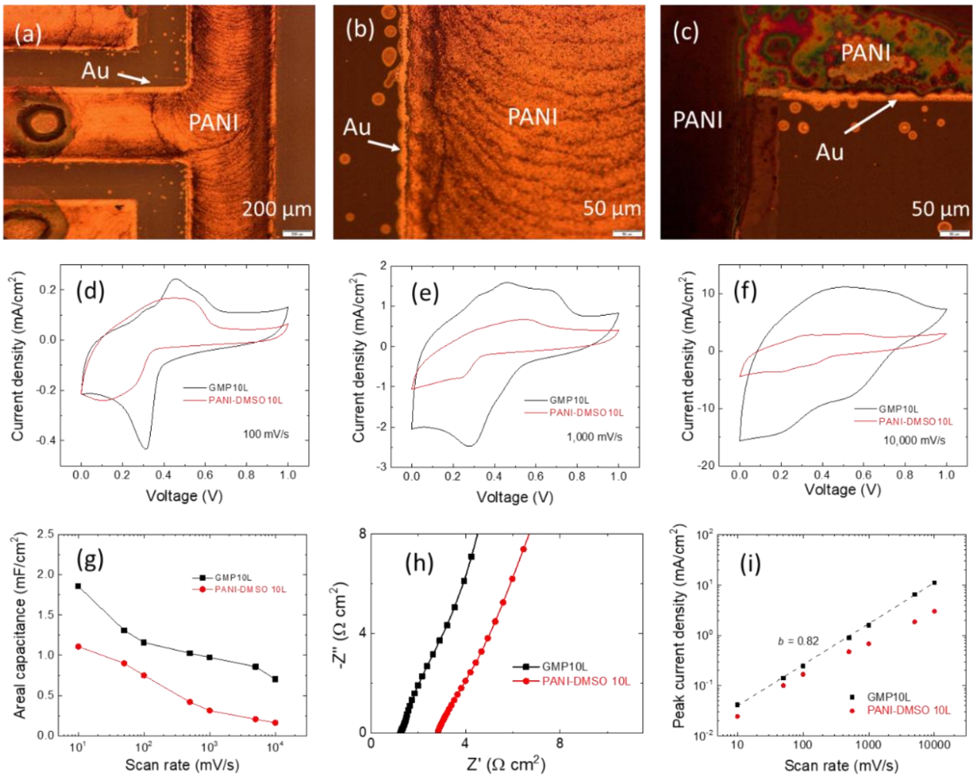

Fig. S8 Characterization of microsupercapacitors with pure PANI electrodes. The devices have almost the same structure as the GMP devices, i.e., inkjet printed gold current collectors and PVA/H_2SO_4/silica gel electrolytes, except that the electrodes are fabricated through inkjet printing of 3 mg mL^{-1} PANI/DMSO dispersions for 10 layers. (**a-c**) Micrographs of the printed PANI microsupercapacitors on glass slides. Different from the globally uniform GMP patterns, the PANI patterns become non-uniform after being overwritten by 10 times. (**d-f**) CV of the PANI and GMP microsupercapacitors at scan rates of 100, 1000, and 10000 mV s^{-1}. (**g**) Dependence of areal capacitance on scan rates, (**h**) impedance spectra and (**i**) fitting of the

peak current density (i_p) versus scan rate (v) by the power law ($i_p \sim v^b$) for the PANI and GMP microsupercapacitors. Although the PANI devices have comparable performance to the GMP devices at low scan rates, they become evidently inferior at high scan rates.

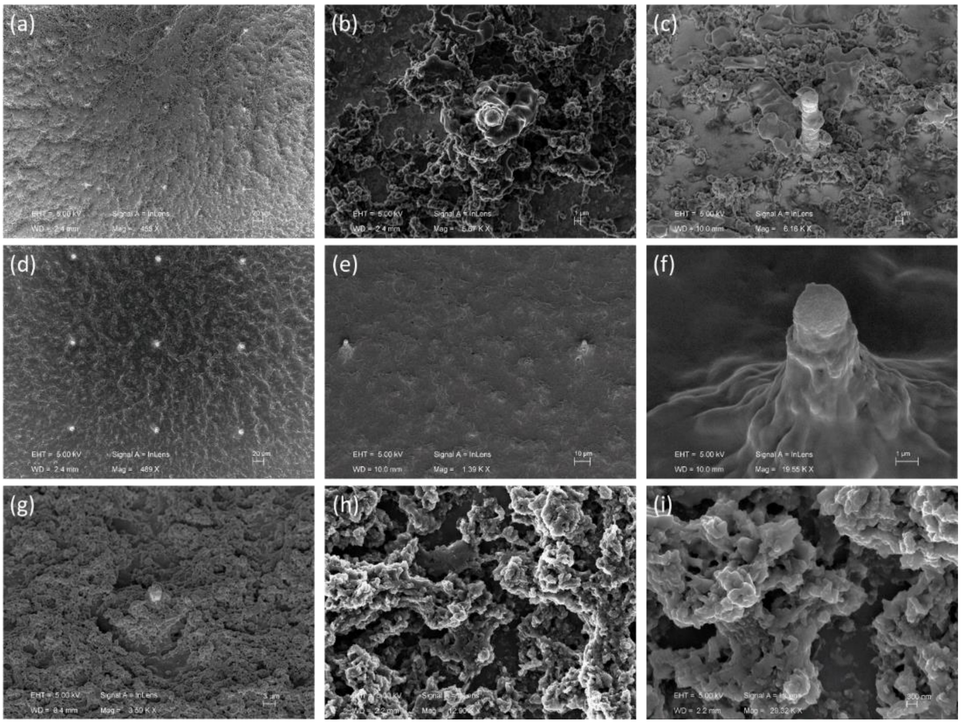

Fig. S9 More SEM images indicating the printing processing of the 3-cycle GMP micro-supercapacitors. (**a-c**) is for the printing of 10 layers of PVA/H_2SO_4/silica gel electrolytes above 40 layers of GMP in the first cycle, corresponding to Fig. S13b. (**d-f**) is for the printing of PEDOT:PSS (40 layers) as current collector for the second cycle, corresponding to Fig. S13c. (**g-i**) is for the printing of GMP (40 layers) as the electrode for the third cycle, corresponding to Figs. 3g and S13g. It is clearly shown that through pores still occur in the printed GMP films on top of the PEDOT:PSS current collectors in spite of much smaller pore size.

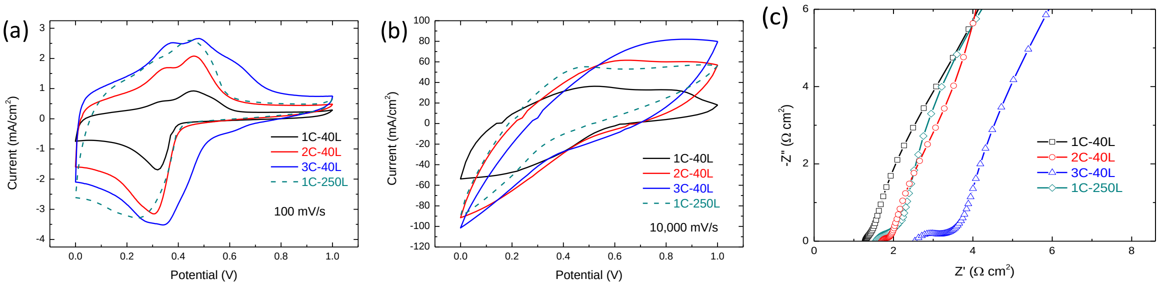

Fig. S10 More electrochemical characterization of the 3D-structured GMP micro-supercapacitors. (**a, b**) CV at scan rates of 100 and 10000 mV s^{-1}. (**c**) Impedance spectra

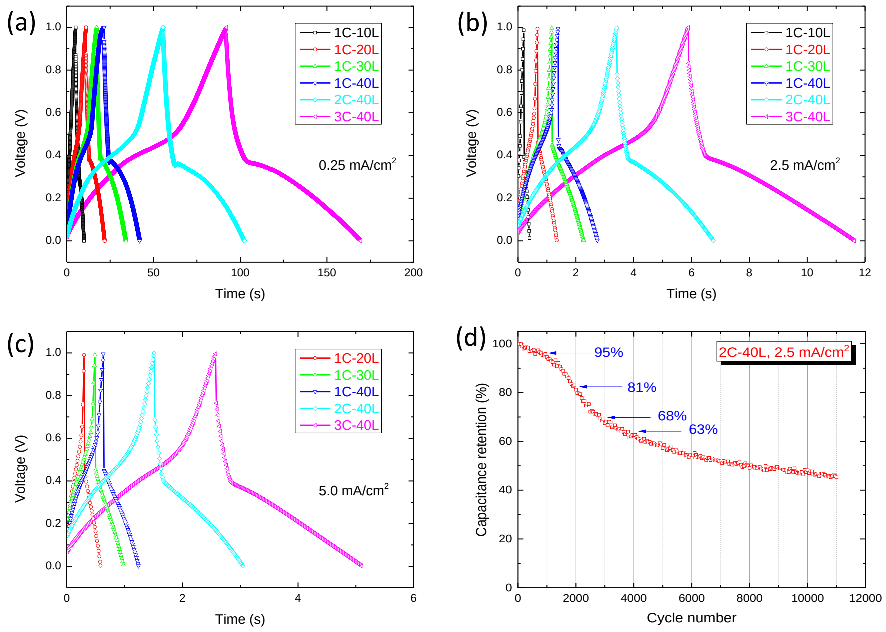

Fig. S11 Galvanostatic charging/discharging (GCD) curves and cyclic performance of the 2D/3D GMP microsupercapacitors. GCD curves of the devices at different current densities of 0.25 mA cm^{-2} (**a**), 2.5 mA cm^{-2} (**b**), and 5.0 mA cm^{-2} (**c**). (**d**) Capacitance retention of a 2-cycle GMP microsupercapacitor tested through the galvanostatic cycling at the current density of 2.5 mA cm^{-2}. The capacitance in (**d**) is calculated through $C = I_D\Delta t/\Delta V$ where I_D is the discharging current, Δt is the discharging time and $\Delta V = 1$ V is the voltage window. The initial 50 cycles were skipped in (**d**).

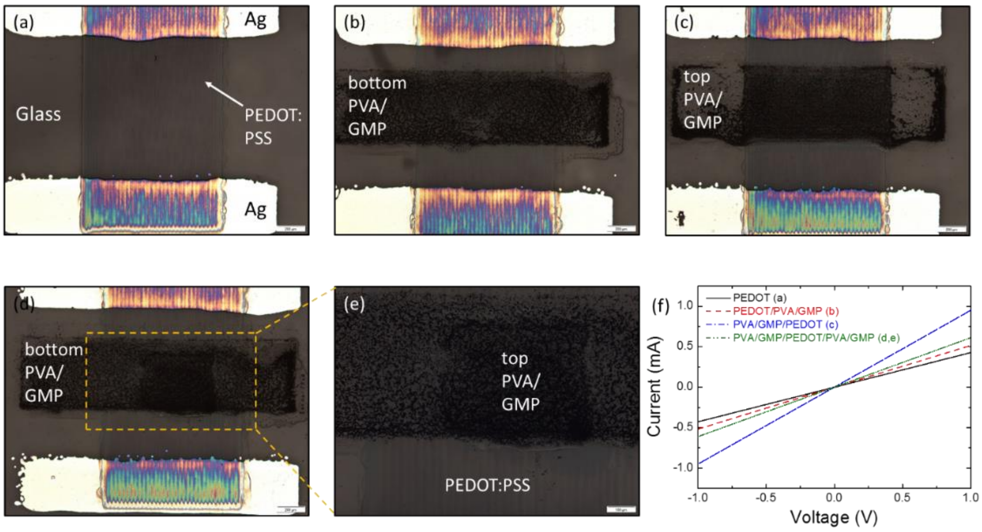

Fig. S12 Electrical characterization of the printed PEDOT:PSS (current collector) films under different integrations with the GMP electrodes and PVA/H_2SO_4/silica gel electrolytes to verify that no conductance degradation occurs during the integrations. (**a**) Micrograph of a printed rectangular **PEDOT:PSS film directly on glass slide**. The PEDOT:PSS film bridges two separate printed silver electrodes for electrical characterization. (**b**) **PEDOT:PSS *above* GMP/PVA layers**. Before printing of PEDOT:PSS, two bottom layers of GMP and PVA gel electrolytes were printed in sequence as a horizontal strip inside the gap between the two silver electrodes. Then, the PEDOT:PSS film was printed above the GMP/PVA layers to

connect the silver electrodes. (**c**) **PEDOT:PSS *underneath* GMP/PVA layers.** PEDOT:PSS was printed first on the glass slide, followed by printing of GMP and PVA layers in sequence on top of PEDOT:PSS. (**d**) **PEDOT:PSS *in-between* GMP/PVA layers.** First, two bottom layers of GMP and PVA gel electrolytes were printed in sequence. Then, PEDOT:PSS was printed above. Finally, two top layers of GMP and PVA (of smaller-area) were printed on top of PEDOT:PSS. (**e**) A close-up view of the top GMP/PVA layers in (**d**). In all the cases, PEDOT:PSS, GMP and PVA were printed with 10 passes, 10 passes and 3 passes, respectively. Scale bars in (**a-d**) are 200 µm, and in (**b**) 100 µm. (**f**) Current-voltage curves of the PEDOT:PSS films under the above four different conditions.

From (**a-e**), one can see the printing of GMP and PVA inks does not deform the PEDOT:PSS layers. In (**f**), As compared with the pure PEDOT:PSS on glass (**a**), the currents increase in all the other three cases (**b-d**), suggesting the integration with GMP and PVA electrolytes actually improves the conductance of PEDOT:PSS, probably because of the doping effects of the ethylene glycol solvent in the GMP ink and sulfuric acid in the PVA electrolytes.

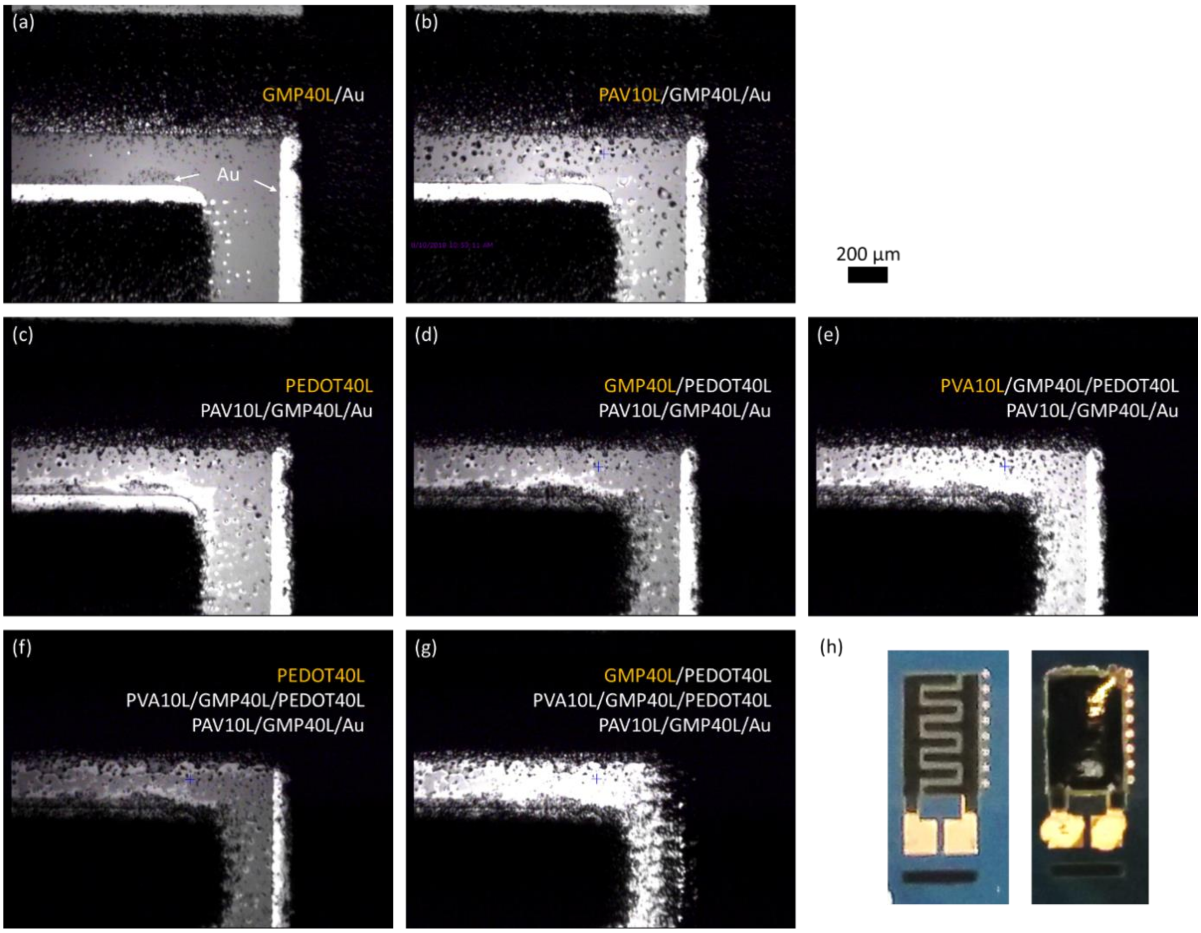

Fig. S13 Evolution of inkjet printing of a 3-cycle microsupercapacitor. (**a-g**) Micrographs focusing on the same region of the device after printing every heterogeneous material layer (as indicated in each sub-Fig.). After being overwritten for >200 passes (in total) of different materials, the device structure is not severely deformed, which enables the direct fabrication of heterogeneous 3D microsupercapacitors simply through inkjet printing. (**a-g**) share the same scale bar in the top-right corner. (**h**) Photographs of the printed 3-cycle device (left, corresponding to **g**) and the final device after drop casting of global PVA electrode and silver paste for electrochemical characterization (right)
